# On the Diagnosis of Urinary Changes

**Published:** 1847-01

**Authors:** 


### BUFFALO MEDICAL JOURNAL

AND

### MONTHLY REVIEW.

VOL. 2. JANUARY, 1847. NO. 8.

### ORIGINAL COMMUNICATIONS.

ART. 1.—*On the Diagnosis of Urinary Changes.* By the EDITOR.

In view of the importance with which late chemical and physiological researches have invested the disorders of the urinary secretion, it has occurred to us that a succinct account of the more prominent circumstances involved in their diagnosis would prove acceptable to our readers. It is convenient for the physician, whose attention must necessarily be directed almost simultaneously to the different departments of practical medicine, to have at hand a synopsis of facts appertaining to this, and other branches of his art, by means of which he can occasionally refresh his memory, and to which he can refer when cases occur requiring their application. It is to subserve these ends that we take the trouble of preparing the following brief summary of the chief circumstances to be borne in mind in interrogating the urine for pathological changes. The claims of urinary disorders, as constituting in themselves a class of interesting affections, but, still more, as furnishing important manifestations, or symptoms of diseases located elewhere, or affecting the system at large, have assumed too important a character to admit of their being overlooked or neglected by practitioners who aim, we will not say to keep pace with, but to follow in the track of pathological progress. The time must soon arrive, when, to omit examination of the urine in the majority of diseases, will be as disreputable as to despise the stethoscope in affections of the lungs and heart. Prudence, therefore, as well as other considerations of a higher and more disinterested character, dictate to every practitioner the necessity of acquiring knowledge of urinary changes, and familiarity with the methods of distinguishing them, sufficient for ordinary practical purposes. This is by no means an intricate and difficult task. Doubtless a false impression on this point repels many from directing their attention to the subject. It is a current belief that to test the various alterations which the urine undergoes, requires minute acquaintance with the details of chemistry, and no small skill in analytical manipulations. This is an entirely erroneous idea. Very little attention and practice will enable the practitioner to determine all the more important facts involved in diagnosis; and the time and trouble which the necessary examinations will demand are but trifling obstacles.

In the following brief synopsis we shall include the rules and methods of analysis which are required to furnish, in most cases, all the information to be desired by the practitioner. The authorities consulted are Prout, Robert Willis, Christison, Bird and Simon. With this general acknowledgment we need not refer to them for the particular facts taken from them severally. The more important disorders of the urine, nosologically arranged as such, are as follows :

1. Aqueous Urine, Hydruria, Diabetes insipidus, Diuresis.
2. Superabundance of Urea, Azoturia.
3. Undue diminution of Urea; Anazoturia.
4. Excess of Lithic Acid and the Lithates; Lithuria, Red Gravel.
5. Excess of the Phosphates, White Gravel.
6. Presence of Oxalic Acid.
7. Diabetes Mellitus or Melituria.
8. Albuminaria, or Bright's Disease.
9. Mucus, Pus, Blood, &c. in the Urine.

We will take up these several classes of changes in the foregoing order, and state briefly the means by which they are severally to be distinguished.

A. *Increase of the Aqueous portion of the Urine.*—This is hardly to be considered a disease. It occurs transiently after free indulgence in alcoholic or other beverages (urina potus,) during mental excitement or anxiety, in some persons from intellectual activity, in hysteria, from a peculiar idiosyncrasy in which at the same time a constant craving for drinks is experienced. The chief advantage of recognising it as a pathological condition is, that it may be distinguished from other affections of a much graver character, which are attended with an increased secretion of urine.

When the quantity of urine is increased, without an increase of the solid constituents, the specific gravity should be correspondingly low. The specific gravity is most readily determined by a hydrometer made for the purpose, called the *urinometer* or *gravimeter*. The quantity of urine passed in 24 hours is to be ascertained, and compared with the average standard, which is from thirty to forty ounces. If the sp. grav. be lessened in proportion to the increase of the quantity, it is presumable that the excess is in the aqueous portion.

To obtain the exact relation of the solid ingredients, collectively, to the whole quantity of urine, the following method is to be pursued : A given quantity is to be carefully evaporated, and the solid residue, after being completely desiccated, accurately weighed. Then, by the arithmetic rule of proportion, we are able to arrive at the precise amount of ingredients in the whole quantity of urine. In order to spare this trouble, the ratio of solid and fluid matter to a given quantity of the whole fluid (1000 grs.) has been calculated for given numbers expressive of the specific gravity, and the results have been thrown into tables, so that having ascertained the specific gravity by the urinometer, on reference to the tables, we can in a moment determine the whole amount of solids contained in the quantity passed in 24 hours, with sufficient accuracy for most practical purposes. [For the table see R. Willis, on urinary diseases; Bird, on urinary deposits; Bell and Stokes' practice.]

Diminution of the aqueous portion of the urine is to be determined conversely in the same manner. This, like the former, is seldom, if ever, a disease. It is present in febrile and inflammatory affections; and, within the limits of health, may be occasioned by increased transudation from the cutaneous surface, and various other circumstances. The specific gravity of healthy urine, it is to be recollected, is about 1,020.

B. *Superabundance of Urea.* — The density of the urine is increased in this disorder, provided there be not at the same time an excess of the aqueous portion. It frequently co-exists with diuresis, forming a species of *Diabetes insipidus.* Hence it is important to ascertain the whole quantity of urine passed in the 24 hours in order to determine the amount of *absolute* increase of the urea. The *relative* excess of urea may be ascertained with sufficient accuracy for ordinary practical purposes in the following manner : A small quantity of urine is to be poured into a watch glass, and half or an equal quantity of nitric acid added, so that the latter will fall to the bottom of the glass; the glass is then to be allowed to float on cold water. If only the normal proportion of urea be present, no striking effect will be produced. If the urea exceed considerably the normal proportion, crystals of the nitrate of urea are shortly observable at the bottom of the glass; or the mixture may become more or less solidified. The degree of excess is to be estimated, in part, by the time occupied in the formation of crystals. This may vary from a few moments to several hours. Healthy urine does not yield cystals in this mode except it be concentrated by evaporation. To ascertain precisely the quantity of urea in a given quantity of urine, a more intricate, but not difficult process is required. For this, Simon's Animal Chemistry may be consulted.

Urine holding an excess of urea is generally straw-colored or pale. It may be brown like porter. It speedily gives off a strong ammoniacal odour, owing to the decomposition of the urea.

This form of disorder is found associated with emaciation and debility, which are otherwise unaccountable. Examination of the urine will therefore frequently throw light on cases which were before obscure.

C. *Diminution of Urea.*—If the urea be diminished, the specific gravity will be correspondingly low, unless diuresis accompany it, which is not unusual. It is therefore, as in the former instance, important to take into consideration the whole quantity of urine passed in the 24 hours, so as to estimate the absolute as well as relative deficiency. The urine under these circumstances acquires on standing a putrid, sour odour compared to cabbage-water. The usual ammoniacal smell is deficient or absent, owing to the smaller quantity of urea to be converted by decomposition into ammonia.

The pathological relations and general symptoms of this form of disorder are but little known. It is doubtless connected with some fault in the processes of assimilation, (primary or secondary) by which the urea is formed.

The disorders relating both to the excess and deficiency of urea are of less frequent occurrence, and practical importance than those which are to follow.

D. *Excess of Lithic Acid and Lithates.*—It is still a question among chemical observers in what form or forms Lithic acid exists in the urine. The majority, however, adopt the opinion of Dr. Prout in opposition to that of Berzelius. Dr. Prout thinks that it does not exists in a free state, but in combination mostly with ammonia, forming the lithate of ammonia. It may exist in combination with soda. Lithic acid and the lithates (ammonia and soda) when present abnormally in the urine, are presented in the form of a sedimentary deposit. Lithic acid is much more rarely observed in an isolated sedimentary form, but it is occasionally presented. It is always in crystals, sometimes large enough to be discerned with the naked eye, but generally requiring the microscope. Their peculiar characters under the microscope are described and figured in the work of Golding Bird. In this synopsis we must omit microscopical appearances of these and other deposits, as they would occupy too much space. The reader is referred for facts relating to this very interesting and valuable department of examination of the urine to the work just referred to. Lithic acid crystals assume different colors between a crimson red and fawn color. They may be set free and studied with the microscope in healthy urine by adding nitric acid. The addition of nitric acid sometimes occasions a copious deposit, which, without care, may be confounded with albumen. The presence of lithic acid crystals does not necessary denote an excess of this urinary principle; for if any acid be superabundant, (muriatic, sulphuric, lactic, &c.,) it may combine with the ammonia already in combination with the lithic, and set the latter free. To determine in doubtful cases whether it be absolutely in excess, the whole urine passed in 24 hours must be collected and measured; a definite quantity must then be tested quantitively for the lithic acid which it contains. By the rule of proportion, the whole amount contained in the whole quantity is readily ascertained. If this exceed considerably the average healthy quantity, which is a fraction over 8 grs. in the 24 hours, it is in excess. For the details of the quantitive analysis, which is not difficult, see work of Bird, or Simon's Animal Chemistry.

The most common form in which it is in excess is in combination with ammonia or soda—constituting what is usually called the *Lithates.* These form a non-crystalized, amorphous, or pulverulent sediment. The sediments of the lithate of ammonia have different colors—red, pink, and sometimes, but rarely, nearly white. The red sediment is familiar as the lateritious or brick-dust deposit. The test of these is sufficiently simple. Urine holding the suspected deposit in a test-tube or common vial is to be held over a spirit lamp or near the fire. If on becoming warm the sediment dissolve and the urine be rendered transparent, it consists of the lithates. If, on the other hand, it is unaffected, or the urine be rendered turbid, the disorder is of a different character. The deposit of the lithates occurs transiently from indigestion, or a common cold. It characterises the termination of fevers and inflammations, and sometimes continues through the course of fevers. It occurs in a marked degree in gout and rheumatism, and in some forms of dyspepsia. In general terms it denotes malassimilation, either primary or secondary, or both, but the specific aberrations upon which it depends are yet to be determined. The lithic acid crystals constitute the concretions called red gravel. They occur in the kidney, and passing into the bladder may constitute a nucleus for stone. The lithic acid diathesis, therefore, with reference to this possible result should be known and remedied.

E. *Excess of Phosphates.*—The saline constituents of the urine are of several kinds, but the phosphates predominate, and for most practical purposes, it is sufficient to include them under this generic term. The more important of the earthy salts are phosphate of ammonia and magnesia, called frequently the triple phosphate; the phosphate of lime; and the carbonate of lime. They are nearly insoluble, unless an acid be present. The ammonio—phosphate of soda is another triple salt which is soluble, and therefore not so easily determined to be in excess. The phosphates in excess (with exception of the salt last mentioned,) manifest themselves by a white sediment, which may from appearance alone be mistaken for the lithates, mucus, pus, or albumen. It is to be tested first by heat. Heat applied in the same way as when testing for the lithates, produces no change, or if any, it increases the deposit. Nitric acid is then to be added, (a few drops will suffice.) If the sediment consists of the phosphates, it will be found to dissolve, and render the urine clear. Urine holding the phosphates in excess is usually pale, frequently resembling whey, and sometimes milky in appearance. In all other disorders of the urine it has an acid reaction upon litmus. In this disorder the acid reaction is faint, or it may be neutral, or it may be decidedly alkalescent. Alkalescent urine necessarily throws down phosphatic deposits, because the phosphates are insoluble if no acid be present. A white deposit occurring in urine presenting an alkaline reaction may therefore be known to consist of the phosphates. To test the acidity of the urine the practitioner should be provided with blue and red test paper.

This disorder is connected with some bad forms of dyspepsia. It generally denotes great loss of constitutional vigor, and the breaking up of the constitution. It is therefore an unfavorable sign. There are, however, striking exceptions to this remark. The phosphatic sediments are amorphous, with the exception of phosphate of ammonia and magnesia. This is presented in minute cystals, which frequently collect on the surface of the urine forming an irridescent scum. Crystals of this salt forming in the kidney constitute what is called white-gravel. Calculi with these crystals for nuclei are very rare. They generally proceed from crystals of lithic acid as nuclei, but the latter frequently increase in size from phosphatic accretion.

F. *Presence of Oxalic Acid.*—The foregoing disorders have related to principles which exist in the urine in a state of health; but oxalic acid is not present in healthy urine. Its existence in ever so small proportion is therefore a mark of disease. It is a rare disorder; but since the investigations of Golding Bird it has been found not to be so extremely infrequent as had been heretofore supposed. It does not usually form a deposit, and its presence is to be determined by the microscope. Its pathological relations are obscure. The oxalite of lime constitutes the rare form of calculus called mulberry calculus.

G. *Presence of Sugar in the Urine. Diabetes Mellitus.*—Sugar does not exist in healthy urine. Until lately it was supposed not to exist in the blood even of diabetic patients. Its presence under the latter circumstances is, however, now well ascertained. Diabetes may be suspected when the quantity is greatly increased, and when it has a high specific gravity. The density of diabetic urine is seldom under 1,025, and may be as high as 1,050. It is usually of a pale color, and froths on agitation more than healthy urine.

There are numerous tests for sugar in the urine, but the two following will suffice for all practical purposes:

1st test. A few drops of the suspected urine are to be placed on a porcelain dish, and carefully evaporated. When dried add a few drops of dilute sulphuric acid, (one part of acid to six or eight of water) and heat gently for a few moments. If the urine contain sugar the spot soon turns black, owing to the carbon of the sugar; otherwise it assumes an orange color. This is an easy and very delicate test.

2d test. Put two teaspoonfuls of yeast into a vial, and pour upon it three or four ounces of the suspected urine. Place the mixture in a warm place. If sugar be present fermentation will soon take place. If sugar be not present it will remain unaffected. A good way to illustrate the significancy of this test is to take three vials each containing the same quantity of yeast. Add to one vial the suspected urine; to another, water holding in solution a few grains of sugar; and to the third, urine not supposed to be diabetic. Place them all together in a warm place. The difference will show the value of the test. An acquaintance with these simple methods of determining the presence of sugar renders tasting quite an unnecessary sacrifice of inclination. To taste of a patient's urine is therefore to be regarded as an evidence of an unusual want of fastidiousness, or a confession of ignorance.

H. *Presence of Albumen. Bright's Disease.*—Albumen is not a principle of healthy urine. Its presence is therefore a sign of disorder. It is occasionally noticed in connection with a variety of diseases; when it is considerable in quantity, and persistent, however, it is a pretty certain criterion of granular degeneration of the kidneys, or Bright's disease. Under these circumstances it coexists with anasarca and other dropsical effusions; and may be associated with numerous other affections. The tests of its presence are heat and nitric acid. If a quantity of urine in a test tube or common vial be heated to near the boiling point, the albumen, if present, is coagulated and precipitated. A suspicion may possibly exist that the deposit thrown down in this way consists of the phosphates. To settle this point a little nitric acid should be added, which, if the phosphates are deposited, will cause them to dissolve; otherwise the deposit will remain. Nitric acid added at first will, also, coagulate and throw down the albumen. This, from appearances alone, may be confounded with deposit of lithic acid, which sometimes occurs on the addition of nitric acid. To obviate this mistake the urine should also be tested by heat; or the white deposit examined with the microscope, which, if it consist of lithic acid, will demonstrate its crystalized form. For all practical purposes it suffices to estimate the relative quantity of albumen by noting how high the deposit extends up the vial or test-tube, after being permitted to stand for several hours.

I. *Mucus, Pus and Blood in the Urine.*—The presence of blood, if in considerable quantity, is easily enough detected by the eye alone. If diffused through the urine in small quantity it is more difficult to be ascertained. The color of the urine is blood red. A white rag dipped in it is colored red. In very doubtful cases the microscope must be resorted to, when the appearance of the blood disks will settle the question.

It is sometimes useful to distinguish pus from mucus. Mucus generally falls to the bottom of the vessel, and when the urine has been gently poured away, remains with greater or less tenacity. If the urine be agitated with the mucus, the latter does not commingle, but appears in the form of long shreds or jagged portions. Pus, on the other hand, is capable of being readily mixed with the urine on agitation; it does not form a viscid tenacious mass, and the supernatant urine cannot so readily be poured away from it. These characters will suffice whenever either exists in any considerable quantities. The microscope, in cases of a doubtful character, furnishes a ready test, the appearance of the pus globule being sufficiently characteristic.

The foregoing synopsis, it is believed contains the necessary rules and methods of testing urine, in so far as it is requisite for the general practitioner to be acquainted with the subject. As we have said, our object in presenting them in this form is, that it may serve to refresh the memory, and for convenience of reference. If it should be made to appear that the subject is not a difficult one, but, on the contrary, only requires a little attention to be understood sufficiently for all practical purposes, the little trouble which this brief summary has cost us may answer another good end—viz., it may convince our readers that there is no good apology for neglecting to avail themselves of the knowledge to be derived from examinations of the urine.

